# Complications of COVID-19 Pneumonia and Multiple Sclerosis Exacerbation

**DOI:** 10.7759/cureus.17506

**Published:** 2021-08-27

**Authors:** Prashanth J Jaisankar, Aurelia Kucera, Christine M Lomiguen, Justin Chin

**Affiliations:** 1 Department of Medical Education, Lake Erie College of Osteopathic Medicine, Erie, USA; 2 Department of Family Medicine, Millcreek Community Hospital, Erie, USA; 3 Department of Family Medicine, LifeLong Medical Care, Richmond, USA

**Keywords:** covid-19, multiple sclerosis, ms, relapsing-remitting multiple sclerosis, multiple sclerosis flare-ups, multiple sclerosis exacerbation, covid-19 pneumonia, dmard, covid

## Abstract

Multiple sclerosis (MS) is the most common autoimmune disease in the United States, in which demyelination of the brain and spinal cord disrupts the transmission of signals throughout the body. With an average life expectancy of 30 years from the start of the disease, treatment relies on symptom management through steroids and disease-modifying agents, as there is no cure. While MS patients have not been shown to be at increased risk for coronavirus disease 19 (COVID-19) infection, prolonged hospitalizations and severe COVID-19 sequelae have been linked to various MS subgroups. Limited studies, however, have reported on the role of COVID-19 in precipitating MS exacerbations, as flare-ups often occur during times of stress or immunological insult. Here we present a 45-year-old patient with relapsing-remitting multiple sclerosis whose neurological symptoms worsened sharply in the weeks following an inpatient admission for COVID-19 pneumonia.

## Introduction

Multiple sclerosis (MS) is the most common autoimmune disease of the central nervous system, with a global prevalence estimated at 2.8 million people (35.9 per 100,000 population), with 914,000 patients in the United States alone [1]. Also known as encephalomyelitis disseminata, it is a demyelinating disease that damages the brain and spinal cord, grossly affecting nerve cell conduction. Symptoms and presentation can be variable as the affected area of the central nervous system dictates its physical manifestation, with Uhthoff's phenomenon (transient worsening of neurological symptoms in heat) and Lhermitte's sign (sudden sensation resembling an electric shock) being pathoanatomic for MS [2]. Typically diagnosed using McDonald’s criteria between the ages of 20 and 50, MS is twice as common in women compared to men [3]. The exact cause is unknown; however, it has been hypothesized that geographical, genetic, and infectious etiologies play a role in MS development [4]. Treatment is often aimed at symptom management with an immunomodulatory or immunosuppressive disease-modifying therapy (DMT), which increases infection risk and hospital admissions related to sepsis/other infectious sequelae, with higher one-month mortality rates post-discharge compared to non-DMT patients [5]. Patients and medical providers often need to balance the risks and benefits of immunosuppression and disease management, which has been further complicated during the coronavirus disease 19 (COVID-19) pandemic.

Coronavirus disease 19 (COVID-19) is caused by the severe acute respiratory syndrome coronavirus 2 (SARS-CoV-2) virus and has caused substantial morbidity and mortality since its discovery in 2019 [6]. In severe COVID-19, multi-organ damage and dysfunction are common, as the immune system produces large, unregulated quantities of cytokines, leading to leukocyte migration and cytokine storm syndrome. While acute respiratory distress syndrome (ARDS) and pulmonary sequelae have been characteristic of severe COVID-19, neurological manifestations have also been well-documented, particularly during the post-recovery phase [7]. Early observational studies indicate that the incidence, risk factors, and outcomes of COVID-19 between MS patients and the general public are similar; it is presently unknown how episodes of severe COVID-19 affect the long-term course of MS or the precipitation of exacerbations [8]. Here we present the case of a 45-year-old patient with relapsing-remitting multiple sclerosis whose neurological symptoms worsened sharply in the weeks following an inpatient admission for COVID-19 pneumonia.

## Case presentation

Mr. J was a 45-year-old Caucasian male with a past medical history significant for chronic kidney disease stage 3a and relapsing-remitting multiple sclerosis (RRMS) in remission who presented to the emergency department (ED) of a rural community hospital with shortness of breath and altered mental status (AMS). Surgical, family, and social history were non-contributory, and he was not on any disease-modifying medications for his MS. Of note, two weeks prior, he was diagnosed with COVID-19-related pneumonia that resulted in a one-week admission for new dysphagia and hypoxia upon testing positive for the presence of the COVID-19 virus. During this prior admission, he received a dexamethasone taper, a five-day course of remdesivir, and supplemental oxygen via nasal cannula. His dysphagia gradually improved and did not require intubation. His hospital course was complicated by mild deconditioning as he was encouraged to bed rest in a prone position but overall responded well to medical treatment. He was weaned off oxygen and discharged home to the care of his family. At discharge, he required a wheelchair, however, appeared to be at his neurological baseline with maintaining short conversations and resumption of a regular diet. He had not received his COVID-19 vaccination during this period.

**Figure 1:**
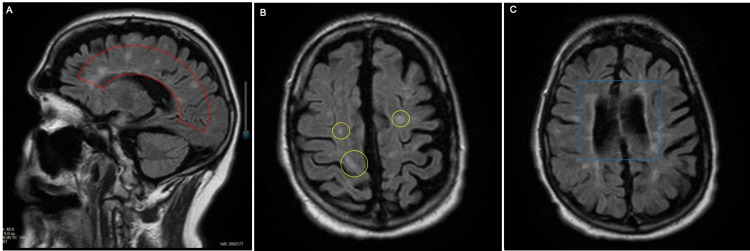
T2-weighted MRI images of the brain. A: Sagittal view showing radiographic findings of Dawson’s fingers in multiple sclerosis (red arc). B: Transverse view with new lesions (yellow circles). C: Transverse view with paraventricular lesions (blue box).

In the following week, Mr. J began to deteriorate according to collateral information from the family in which he became more withdrawn and fatigued. At the ED, he was nonverbal and lethargic, only briefly opening his eyes when his name was called and intermittently groaning throughout the physical exam, which was largely benign; however, was only oriented to name. Computed tomography (CT) of the abdomen and pelvis showed chronic bilateral hydronephrosis; blood urea nitrogen (BUN) and creatinine were significantly elevated from the patient's baseline (within normal limits), at 104 and 3.96, respectively. Magnetic resonance imaging (MRI) of the brain showed new hyperintense periventricular T2 lesions when compared to an older MRI seen in legacy electronic medical records (Figure 1). He was admitted to the medical-surgical floor for acute chronic kidney injury and likely multiple sclerosis exacerbation in the setting of this recent COVID-19 infection.

The patient’s MS exacerbation was treated with methylprednisolone and his fluids were repleted given standard protocol. Urine cultures obtained from the ED grew *Pseudomonas aeruginosa,* and renally adjusted intravenous cefepime was started due to concern for urinary tract infection (UTI) as a cause of his AMS. Complete blood count revealed anemia secondary to chronic kidney disease, with a hemoglobin level of 6.8, for which three units of packed red blood cells were transfused. Over the following three to four days, his mental status and kidney function continued to improve, with down-trending BUN and creatinine as well as measurable urine output. On hospital day five, Mr. J was found to be febrile, with a rectal temperature of 103.1 F, with vitals significant for tachycardia, tachypnea, and diaphoresis. He was transferred to the intensive care unit for closer monitoring, where he was placed on bilevel positive airway pressure (BiPAP). Subsequently, computed tomography angiogram (CTA) showed multiple pulmonary emboli, and the patient was anticoagulated with enoxaparin. Following three days in the ICU, the patient improved clinically with a return to baseline mental status of speaking in short sentences, improved renal function, and decreased oxygen requirements, allowing for transfer to the medicine floor.

The following day, his renal function worsened, secondary to his chronic kidney disease (CKD) and chronic dehydration due to dysphagia. A percutaneous endoscopic gastrostomy (PEG) tube to assist in feeding and hydration was discussed and placed after consent. The patient was discharged home to family once stabilized; however, he presented to the ED two days later, again with altered mental status and BUN/creatinine values suggesting volume depletion. Blood cultures taken in the ED later revealed pseudomonal sepsis, likely secondary to his pre-existing pseudomonal UTI. With prior UTIs noted in outpatient documentation, his current presentation was thought to be a neurogenic bladder in the setting of MS. A suprapubic catheter was placed and a 10-day course of oral ciprofloxacin was prescribed.

On outpatient follow-up two weeks after discharge, Mr. J continued to experience ongoing dysphagia, decreased ambulation, and speaking only in short sentences. He has proceeded with home physical therapy and is waiting for outpatient speech/swallow therapy. He has continued to decline any disease-modifying medications for his MS.

## Discussion

Evidence regarding the clinical course of COVID-19 in patients with MS is limited as the majority of research has been geared toward life-sustaining measures and vaccination efforts. Early case reports have suggested that patients with pre-existing chronic neurological conditions and COVID-19 may develop exacerbation of their neurological symptoms [9]. As noted in the patient’s history, he has relapsing-remitting multiple sclerosis (RRMS), which is the most common MS disease course that is characterized by clearly defined attacks of new or increasing neurological symptoms (which can vary based on the affected part of the central nervous system). The initial diagnosis was established using McDonald's criteria (Table 1). Exacerbations are typically followed by periods of partial or complete recovery (remissions), in which the neurological symptoms disappear, or some may persist and become the new baseline. It is unclear whether COVID-19 infection in RRMS patients can induce exacerbations that result in the development of new neurological baselines or if remission time is impacted. A more recent analysis of cross-sectional data from a registry of MS patients with COVID-19 infection found that ambulatory disability, recent corticosteroid use, older age, male sex, and hypertension were associated with increased clinical severity and had longer recovery periods [10]. While one study found that the incidence of COVID-19 was higher in the studied cohorts compared to the general public, other studies have found that MS patients have similar incidence, risk factors, and outcomes of COVID-19 [11]. While evidence regarding the impact of MS on the clinical course of COVID-19 continues to accumulate, the effect of COVID-19 on the severity and precipitation of MS exacerbation remains an open question.

**Table 1:** Standard criteria used for diagnosis of MS. Dissemination in space refers to one T2 lesion or more in at least two MS typical CNS regions (periventricular, juxtacortical, infratentorial, or spinal cord). Dissemination in time can refer to simultaneous asymptomatic contrast-enhancing/non-enhancing lesion at any time, a new T2/contrast-enhancing lesion on MRI, or a second MS attack/exacerbation. CSF, cerebrospinal fluid; MS, multiple sclerosis

McDonald’s criteria for multiple sclerosis diagnosis		
MS attacks/exacerbation	MS lesions (on imaging)	Additional criteria needed
2 or more	2 or more	None, clinical evidence
2 or more	1	Dissemination in space
1	2 or more	Dissemination in time
1	1	Dissemination in space and time
0 (none from initial onset)	Unclear	One year of disease progression and one of the three: dissemination in space for brain or spinal cord, or positive CSF

In the case of Mr. J, a causal link between the episode of COVID-19 and the subsequent sharp decrease in his neurological function cannot be definitively proven, despite the temporal link between the episode of COVID-19 and the MRI evidence of new demyelination occurring shortly thereafter. Mechanisms by which COVID-19 could potentially cause neurological symptoms include direct viral infection of brain parenchyma, cytokine storm recruiting leukocytes to neural tissue, or causing a prothrombic state resulting in infarction of neural tissue, especially in the context of a disrupted blood-brain barrier of advancing MS [12]. Nevertheless, one neuropathological study of a deceased MS patient with COVID-19 found that there was no evidence for MS disease exacerbation or lesion (re)activation; in fact, even though the decedent had damage to the blood-brain barrier, there was no evidence of SARS-CoV-2 RNA in the MS lesions and adjacent brain parenchyma of the patient [13]. While non-specific to COVID-19, stress and infection are among the leading causes of RRMS exacerbations, such that it is unsurprising that COVID-19 would be an infectious precipitant. Greater studies are needed to determine if MS exacerbations occur more frequently during or after COVID-19 infection.

Even if there is not a direct causal link between COVID-19 and MS exacerbation, Mr. J was left with diminished functional status after his initial discharge from his hospitalization for COVID-19 pneumonia, leaving him more vulnerable to further diminution of his functional status from subsequent MS exacerbation. Immunosuppression with corticosteroids produces a dose-response risk by which the higher the prescribed dose over time, infection risk proportionally increases [14]. Although not applicable to this case, RRMS is also treated long term by multiple disease-modifying medications such as interferons, glatiramer acetate, mitoxantrone, natalizumab, and fingolimod in which there are limited studies on the impact such medications may have relative to COVID-19 infection risk and whether they may be helpful in inducing MS remission [15]. Moreover, since COVID-19 pneumonia and MS exacerbations are treated with moderate-to-high doses of corticosteroids, the patient may have been more vulnerable to infection resulting in his urosepsis. The timeline for RRMS remission and stabilization can range from weeks to months, in which the role of COVID-19 in prolongation or related sequelae is still relatively unknown. Greater research is needed to better understand how COVID-19 can impact the medical and social determinants of health for MS patients, which can include targeted increases in vaccination efforts and recognition of COVID-19 as a potential precipitant to MS exacerbation, thereby leading to possible future areas for improved prevention and further advocacy.

## Conclusions

This case highlights the potential of the pathophysiological processes of COVID-19 and multiple sclerosis to interact in complementary ways, leaving patients uniquely vulnerable to derangements in their functional mental and physical status. Although preliminary evidence indicates that patients with multiple sclerosis who face COVID-19 have risk factors, incidence, and outcomes similar to the general public, more research is needed to determine how COVID-19 affects the course of MS. MS patients who suffer severe COVID-19 are uniquely vulnerable to further deterioration of functional status and potential for MS exacerbation in the post-recovery period.
